# Assembly–Energetics–Control (AEC) Design Framework for Rotary DNA Nanomachines

**DOI:** 10.1002/cbic.70344

**Published:** 2026-04-28

**Authors:** Xue‐Yan Wang, Yicheng Heng, Julian A. Tanner, Simon Chi‐Chin Shiu

**Affiliations:** ^1^ School of Biomedical Sciences Li Ka Shing Faculty of Medicine The University of Hong Kong Hong Kong P. R. China; ^2^ Materials Innovation Institute for Life Sciences and Energy (MILES) HKU‐SIRI Shenzhen P. R. China; ^3^ Advanced Biomedical Instrumentation Centre Hong Kong Science Park Hong Kong P. R. China; ^4^ School of Biomedical Engineering The University of Hong Kong Hong Kong P. R. China; ^5^ Department of Biological Engineering Massachusetts Institute of Technology Cambridge Massachusetts USA

## Abstract

Rotary DNA nanomachines harness programmable DNA assemblies to mimic biological motors that drive cellular energy conversion and mechanical force transmission. With structural designs and actuation mechanisms growing ever more varied, the field faces rising conceptual complexity. To address this, we introduce an engineering‐inspired Assembly–Energetics–Control (AEC) design framework that classifies rotary DNA nanomachines along three independent axes: Assembly (+: Modular / –: Global), Energetics (+: Active / –: Passive), and Control (+: Autonomous / –: Nonautonomous). This triad captures the interplay among these dimensions and overall performance. By mapping representative examples across the AEC framework and identifying underexplored categories, it offers researchers clear guidance for creating rotary DNA nanomachines that truly achieve scalability, autonomy, and biocompatibility.

## Graphical abstract

Rotary DNA nanomachine is an emerging research interest using DNA as building blocks to fabricate nature‐mimicking mechanisms. Here, we suggest a three‐dimensional conceptual framework to categorize the existing designs and identify underexplored directions. This concept serves as a tool to help researchers make decisions in choosing appropriate designs for the corresponding purposes.

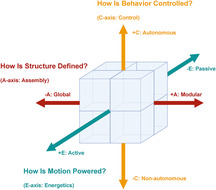

## 1 Introduction

Rotation is one of the most fundamental and efficient forms of mechanical motion in nature, capable of achieving directional conversion and transmission of energy. Compared with linear motion, rotation is controllable for continuous mechanical output within a defined space. Biological molecular motors convert chemical energy into precise and controllable rotational motion, enabling critical life processes within cells, such as energy conversion (e.g., ATP synthase) and targeted transport (e.g., flagellar motors). Inspired by this, the fields of synthetic biology and nanotechnology have recently focused on developing artificial rotating nanomachines, aiming to achieve biomimetic functions in areas such as drug delivery and nanomanufacturing.

Traditional nanomaterials have significant limitations in constructing biomimetic rotating machines. For example, gold nanorods lack programmable motion control, polymer gels struggle to achieve sub‐micron precision, and carbon nanotubes are limited by poor biocompatibility. In contrast, DNA achieves sub‐nanometer precision structural customization through sequence‐specific base pairing, a concept pioneered by Seeman in the early 1980s [1], generating controllable rotational motion through diverse driving mechanisms such as chain displacement reactions, ion gradients, or enzymatic catalysis, and possesses natural biocompatibility [2, 3, 4]. Natural rotating motors, such as ATP synthase, are mostly nucleic acid‐protein complexes themselves, and DNA nanostructures best reproduce the original biomolecular recognition mechanisms and energy conversion pathways. Furthermore, rotary DNA nanostructures can be used to construct single devices or assembled into more complex mechanical systems to work collaboratively with other components. However, as designs diversify, a unified framework is essential to systematically balance assembly strategies, energy sources, and control mechanisms.

**SCHEME 1:**
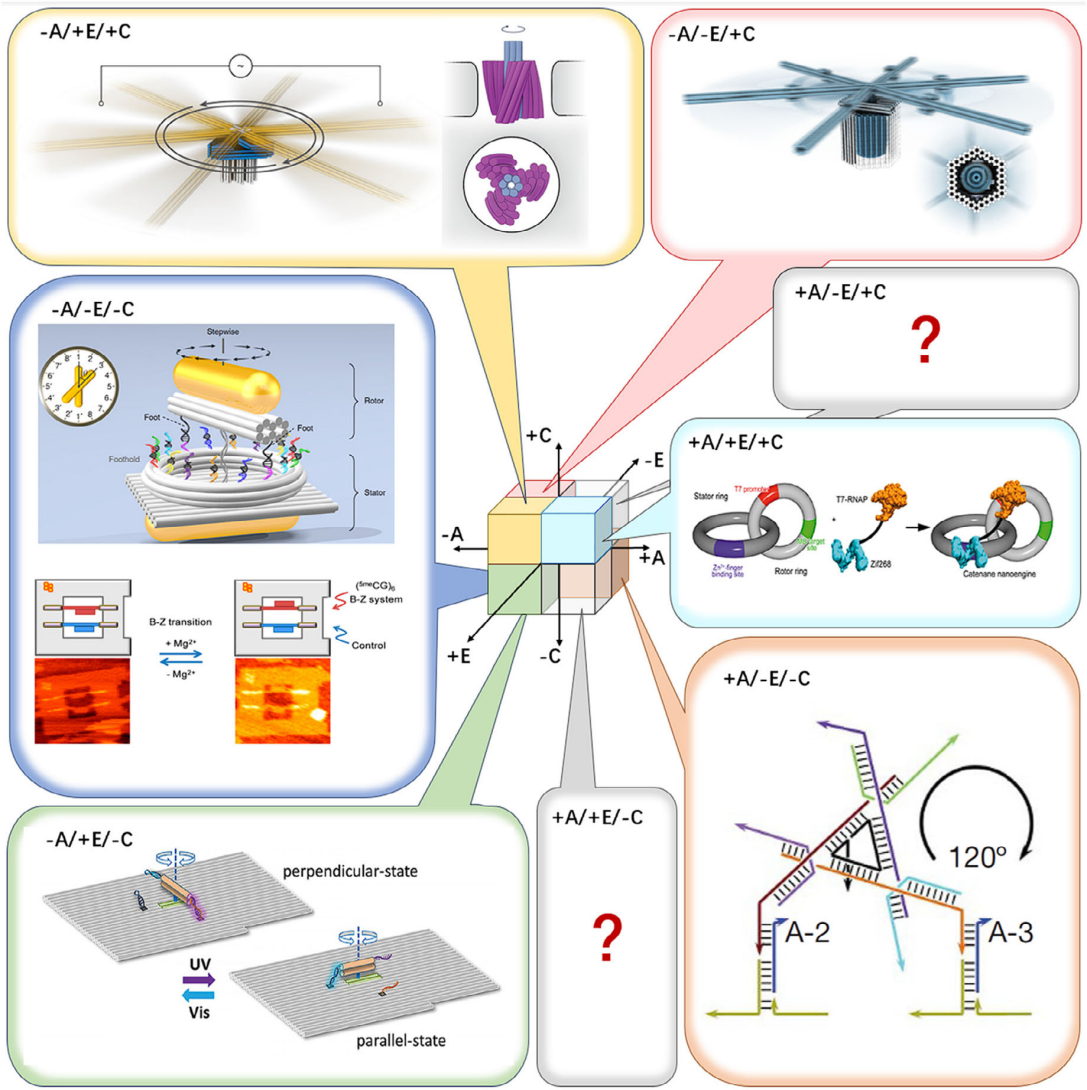
The Assembly–Energetics–Control (AEC) framework for rotary DNA nanomachines. Designs are classified along the three orthogonal axes of Assembly (+: Modular/–: Global), Energetics (+: Active/–: Passive), and Control (+: Autonomous/–: Nonautonomous). This framework identifies underexplored categories of designs +A/+E/–C and +A/–E/+C.

We therefore introduce an engineering‐inspired [5, 6] Assembly–Energetics–Control (AEC) framework to provide unified specifications of different designs (Scheme 1). Far beyond mere classification, AEC forges conceptual bridges among structures, energies, and functions. By positioning rotary DNA nanomachines within a space defined by their assembly strategy, energetic mode, and control logic, this framework enables a systematic understanding of design trade‐offs and helps to reveal unexplored, potentially synergistic regions of the molecular design landscape [7, 8, 9].

## 2 The Three Core Dimensions of Design

To rationalize the rapidly diversifying designs of rotary DNA nanomachines, the AEC framework decomposes their operation into three orthogonal yet interdependent dimensions: Assembly, Energetics, and Control. Each dimension captures a fundamental design choice that governs structural organization, energy transduction, and regulatory logic, respectively. Importantly, these dimensions are not intended as binary classifications. Rather, each axis represents a continuous design spectrum, with the positive (+) and negative (–) poles denoting idealized reference limits that anchor conceptual extremes. Many reported systems occupy intermediate or hybrid positions along one or more axes, reflecting graded integration of modular and global architectures, mixed energetic regimes, and partially autonomous control strategies. The AEC framework thus defines a continuous three‐dimensional design space, within which the eight octants serve as interpretive landmarks rather than rigid categories, enabling systematic comparison while accommodating the inherent complexity of molecular machine design.

### 2.1 How is Structure Defined? (A‐Axis: Assembly)

The scaffold architecture of a rotary DNA nanomachine determines its mechanical rigidity, torque transmission, and geometric precision [10, 11]. Along with the A‐axis (Assembly), architectures differ by their degree of modularity and structural integration. In the AEC framework, the A‐axis addresses an engineering gain‐of‐function: organizational complexity, hierarchical composition, or reconfigurability.

- Modular (+A) designs are composed of small, repetitive DNA building blocks such as tiles, bricks, or polyhedral units, which self‐assemble through local hybridization rules [4, 12, 13]. Such systems offer scalability and flexible design but are susceptible to propagation and precision.
- Global (–A) architectures, exemplified by DNA origami [14], employ a long scaffold strand folded by numerous short staples to form predefined geometries with Nanometer‐scale alignment fidelity [15]. This strategy provides excellent structural coherence and mechanical stiffness but requires intensive sequence optimization and reduces modular adaptability.

Between these two poles, hybrid assemblies integrate modular subunits into global frameworks to balance scalability and rigidity [7]. The A‐axis is therefore a spectrum of examples from modular (+A) to global (–A) structural designs, guiding the decisions on precision, scalability, and mechanical integration.

### 2.2 How is Motion Powered? (E‐Axis: Energetics)

The E‐axis (Energetics) describes the mechanism of energy transduction—whether motion arises passively from thermal conduction or is actively driven by a nonthermal energy source.

- Active (+E) systems convert external energy into directional mechanical work, driven by enzymatic catalysis, photoactivation, potential difference, or ionic gradients [3, 16]. Such systems maintain persistent rotation or cyclic operation through continuous power transduction, often require nonequilibrium conditions and coupled reaction networks [17, 18].
- Passive (–E) systems operate toward thermal equilibrium. As unidirectional rotation cannot be achieved solely by ambient heat, these systems utilize Brownian motion coupled with energy rectification to achieve biased rotation [2, 19]. In these designs, specific chemical energy biases guide the nanomachine to select its mechanical states from random thermal fluctuations. These biases originate either from strand displacement reactions or from structural transitions triggered by environmental stimuli, such as variations in pH and ionic concentration [18].

The E‐axis thus aligns thermally rectified (–E) to actively powered (+E) operation modes, clarifying the trade‐offs between energy efficiency, biocompatibility, and force generation capacity. From an engineering perspective, this axis strictly defines the thermodynamic power transduction driving the system, conceptually independent of how its functional logic is controlled. Navigating this spectrum clarifies the crucial trade‐offs among energy efficiency, biocompatibility, and force generation capacity, while also accommodating systems that operate in intermediate regimes, such as those relying on episodic energy inputs or partially coupled reaction networks.

### 2.3 How is Motion Controlled? (C‐Axis: Control)

The C‐axis (Control) defines how the motion of rotary DNA nanomachines and their function are regulated, ranging from external operation to autonomous regulation [17, 18]. The positive pole (+C) indicates the increase in autonomy, where feedback and logic are programmed within the structural design.

- Autonomous (+C) systems exhibit closed‐loop behavior, maintaining continuous or cyclic motion through internal feedback mechanisms or chemical asymmetry [3, 16]. Examples include enzymatic motors with sustained catalysis and strand‐displacement cascades with intrinsic signal propagation or inhibition loops.
- Nonautonomous (–C) systems function under open‐loop control, executing externally timed operations such as the sequential addition of fuel strands or photoactivated conformational changes [18, 20]. This allows precise temporal manipulation depending on experimental intervention.

Most existing systems occupy intermediate positions between full autonomy and external signal. Rooted in classical engineering principles, the framework intentionally decouples this control dimension from energetics to resolve their frequent conceptual overlap. Essentially, energetics dictates the thermodynamic power transduction, whereas control governs the cybernetic logic and regulatory feedback. This fundamental decoupling reveals that a nanomachine can be actively powered yet remain dependent on step‐by‐step external triggers for operation, or conversely, rely purely on passive thermal fluctuations while achieving continuous autonomous rotation through built‐in structural asymmetry. The control axis likewise represents a gradual transition from nonautonomous (–C) to self‐governing (+C) operation, with numerous designs exhibiting mixed or partially autonomous logic.

## 3 Mapping and Advancing Rotary DNA Nanomachine Design

**FIGURE 1:**
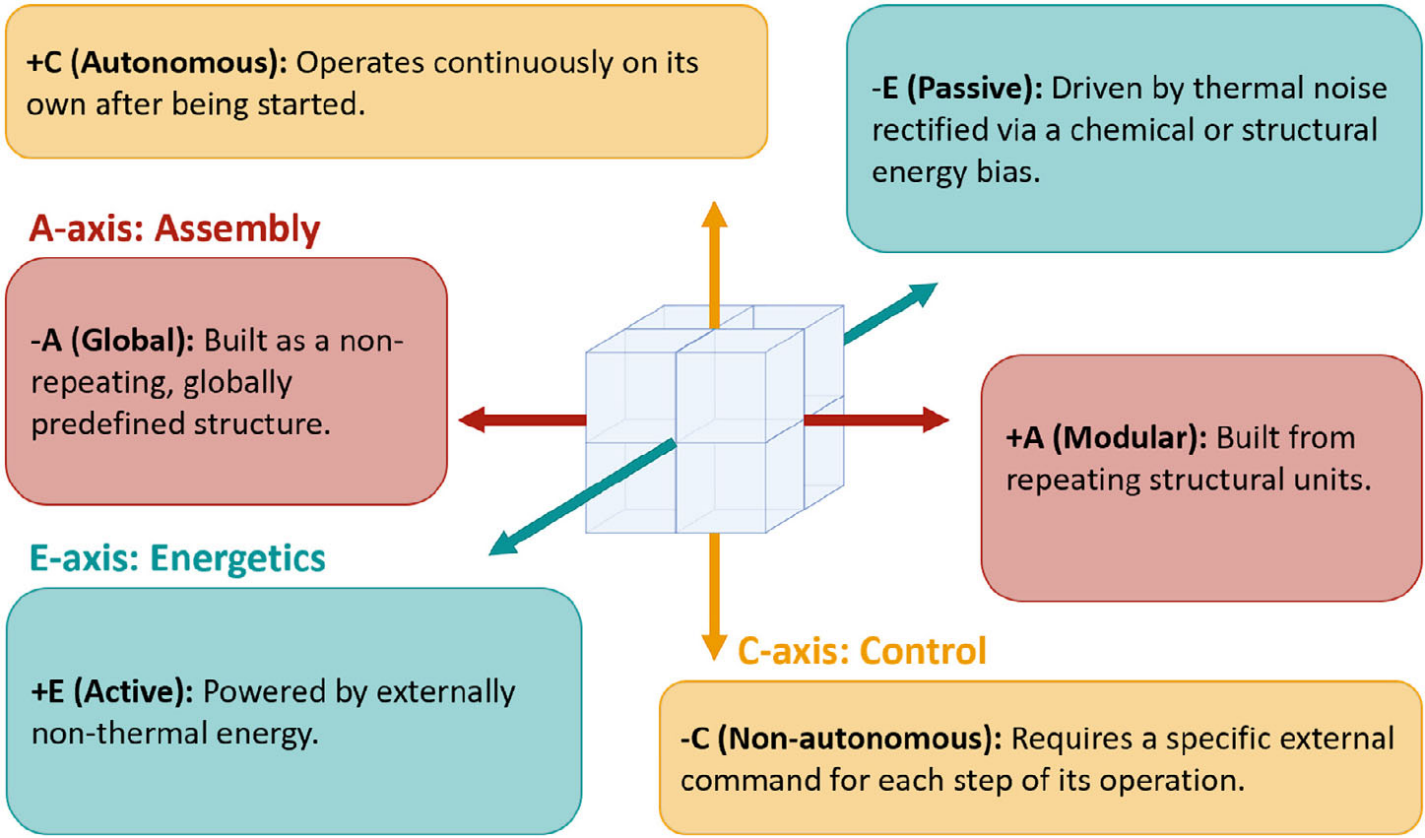
Definition the three core axes of the AEC framework for rotary DNA nanomachines.

The AEC framework organizes rotary DNA nanomachines along the three orthogonal axes: Assembly (+: Modular / –: Global), Energetics (+: Active / –: Passive), and Control (+: Autonomous / –: Nonautonomous) (Figure 1). The necessity for this framework stems directly from the field's conceptual fragmentation. As architecture diversifies, incompatible terminologies make design logic rarely comparable. The AEC framework addresses this by providing a unified language to compare systems based on their foundational engineering principles, rather than isolated features. By mapping existing designs into this 3D design space, we can systematically identify functional trade‐offs, synergistic patterns, and unexplored regions.

**FIGURE 2:**
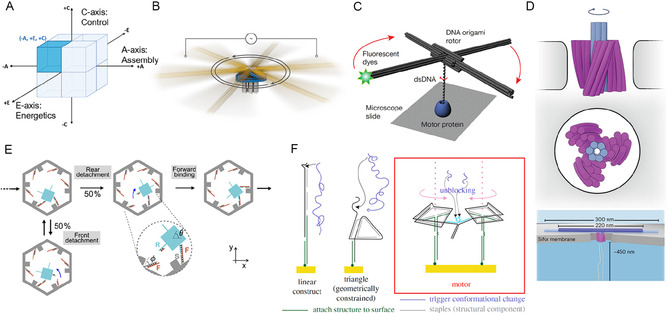
Representative examples of the ‐Global (–A) / +Active (+E) / +Autonomous (+C) octant within the AEC Framework. (A) AEC Framework Conceptual Diagram: A cubic representation highlighting the three orthogonal axes of the AEC framework (Assembly, Energetics, Control). The highlighted area indicates the –A, +E, +C octant. (B) Pumm et al. (2022) [15] Design: A DNA origami (–A) rotary ratchet comprising a fixed pedestal and long rotor arm, autonomously (+C) performs processive unidirectional rotation under an external alternating current electric field (+E), using a Brownian ratchet mechanism to generate torque comparable to F_1_F_0_‐ATPase. (Reproduced with permission from Ref. [15], Copyright: Nature (2022)). (C) Kosuri et al. (2019) [21] Design: A Global (–A) DNA origami‐based rotor for single‐molecule tracking of enzyme‐induced DNA rotation, powered by enzymatic ATP hydrolysis for Active (+E) unwinding and translocation, achieving Autonomous (+C) millisecond‐resolution observation of single‐base‐pair rotational steps. (Reproduced with permission from Ref. [19], Copyright: Springer Nature (2019)). (D) Shi et al. (2024) [16] Design: A DNA origami (–A) turbine with chiral blades anchored in a nanopore, autonomously (+C) rotates unidirectionally (up to 10 rev/s) under transmembrane potential (+E), with rotation direction dictated by blade chirality. (Reproduced with permission from Ref. [7], Copyright: Nature (2023)). (E) Ito et al. (2024) [9] Design: A Global (–A) DNA origami‐based rotary motor with structural asymmetry inducing kinetic bias, powered by nicking enzyme catalysis for Active (+E) energy transduction from fuel DNA cleavage, achieving Autonomous (+C) unidirectional and processive rotation with scalability via multiple connections. (Reproduced with permission from Ref. [12], Copyright: Biophysical Society (2024)) (F) Dunn et al. (2017) [22] Design: A topology‐based (–A) rotary DNA nanomotor formed from two squares, designed for autonomous (+C) unidirectional rotation driven by sequential strand‐displacement reactions that generate active (+E) torque once a passive/active trigger releases the brake. (Reproduced with permission from Ref. [20], Copyright: Royal Society (2017)).

The mapping immediately reveals the dominance of Global (–A) architectures, which leverage the high precision of DNA origami. This structural rigidity is often coupled with Active (+E) power and Autonomous (+C) control to achieve high‐performance, continuous operation. These biological motors achieve remarkable performance by operating at hundreds of revolutions per second with near‐perfect energy efficiency. In contrast, current synthetic DNA machines typically function at much lower millihertz to hertz frequencies, highlighting a significant performance gap that future designs must address. For example, as shown in Figure 2, Pumm et al.'s electric‐field‐driven ratchet (Figure 2B) delivers high‐torque autonomy [15], while Shi et al.'s turbine (Figure 2D) uses ion gradients for waste‐free autonomous rotation [16]. Within this same –A/+E/+C octant, other remarkable examples include Kosuri et al.'s enzyme‐tracked rotor (Figure 2C), Ito et al.'s nuclease‐powered motor (Figure 2E), and Dunn et al.'s topology‐based motor (Figure 2F), all illustrating diverse active actuation mechanisms under autonomous control. While such octant assignments reflect dominant operational tendencies, some systems incorporate mechanistic features from multiple AEC regimes. For instance, Dunn et al.'s autonomous rotary motor combines hybrid assembly, intermittently driven energetics, and conditionally autonomous control, placing it between several AEC extremes despite its overall –A/+E/+C classification.

**FIGURE 3:**
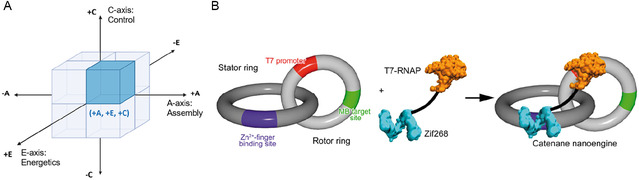
Representative Examples of the +Modular (+A), +Active (+E), +Autonomous (+C) octant within the AEC Framework. (A) AEC Framework Conceptual Diagram: A cubic representation highlighting the three orthogonal axes of the AEC framework (Assembly, Energetics, Control). The highlighted area indicates the +A, +E, +C octant. (B) Valero et al. (2018) [3] Design: A Modular (+A) bio‐hybrid DNA rotor/stator nanoengine that utilized T7 RNA polymerase (RNAP) to consume nucleoside triphosphates as the Active (+E) energy source, achieving fully Autonomous (+C) unidirectional rotary motion. (Reproduced with permission from Ref. [4], Copyright: Springer Nature (2018)).

In contrast, Modular (+A) systems prioritize scalability and hierarchical assembly, exemplified by the autonomous, biocompatible motion of Valero et al.'s enzymatic nanoengine [3] (Figure 3B). Hybrid systems, occupying intermediate positions, further demonstrate the framework's utility in capturing design versatility.

**FIGURE 4:**
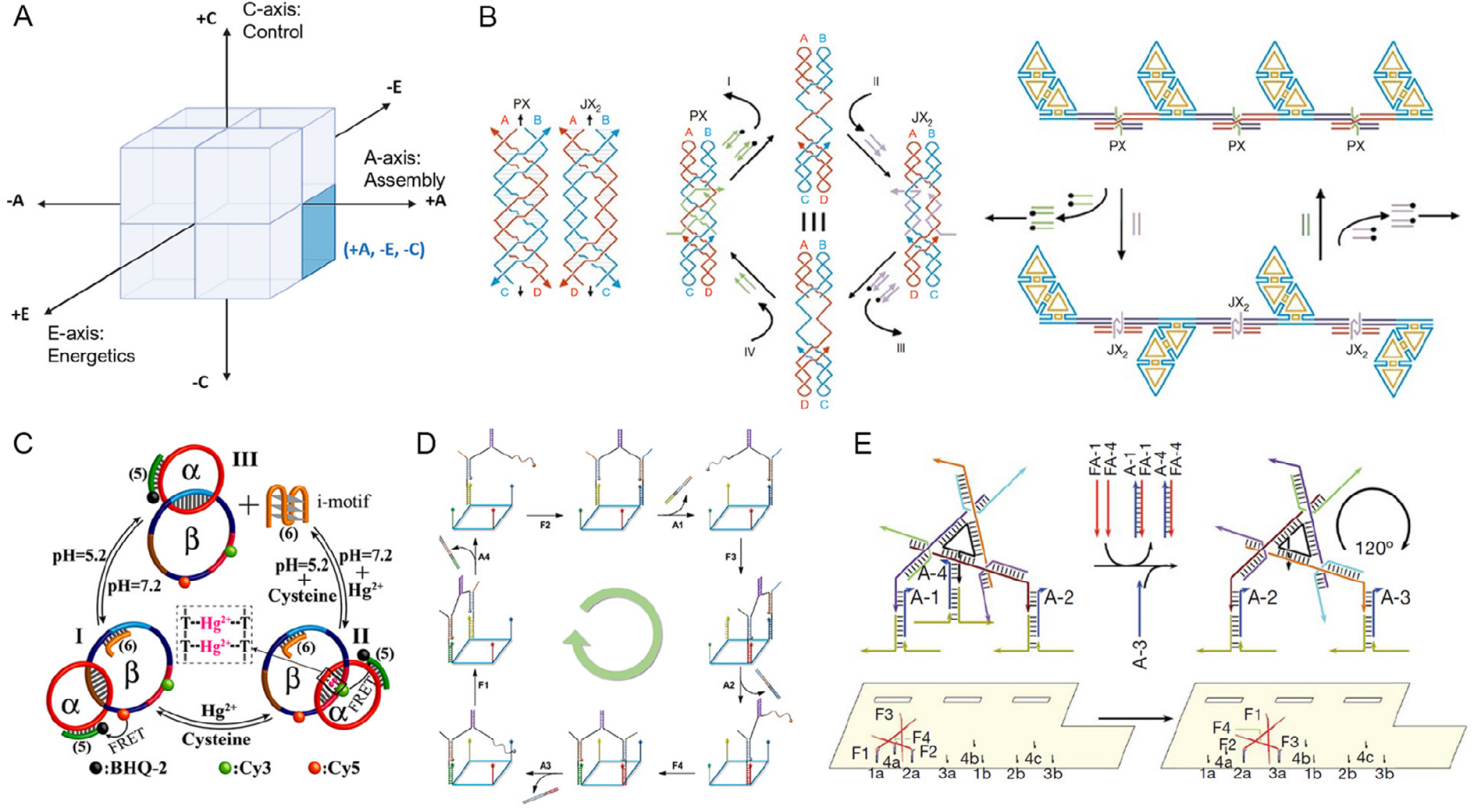
Representative Examples of the +Modular (+A) / ‐Passive (–E) / ‐nonautonomous (–C) octant within the AEC Framework. (A) AEC Framework Conceptual Diagram: A cubic representation highlighting the three orthogonal axes of the AEC framework (Assembly, Energetics, Control). The highlighted area indicates the +A, –E, –C octant. (B) Yan et al. (2002) [23] Design: A Modular (+A) DNA rotary device based on Paranemic crossover‐Juxtapose crossover_2_ hybridization topology switches, powered by strand displacement for Passive (–E) conformational changes, under nonautonomous (–C) control via sequential addition of set and fuel strands, achieving 180° rotary motion. (Reproduced with permission from Ref. [23], Copyright: Springer Nature (2002).) (C) Lu et al. (2013) [24] Design: A Modular (+A) DNA catenane rotary motor translocating across three states, where the rotation is triggered by pH or Hg^2+^/Cysteine inputs in a Passive (–E), nonautonomous (–C) manner. (Reproduced with permission from Ref. [25], Copyright: ACS (2013)). (D) Wang et al. (2017) [25] Design: A Modular (+A) assembly of DNA tetrahedrons into circular stators for a rotary DNA nanomotor with a two‐legged rotor, powered by strand displacement for Passive (–E) locomotion, under nonautonomous (–C) control via sequential addition of fuel and anti‐fuel strands, achieving multicycle circular rotation. (Reproduced with permission from Ref. [26], Copyright: Oxford University Press (2017)). (E) Gu et al. (2010) [26] Design: A DNA tile‐based (+A) nanoscale assembly line featuring a tensegrity‐triangle walker on a prescribed track, powered by strand displacement for Passive (–E) locomotion and cargo transfer, under nonautonomous (–C) control via sequential addition of fuel strands to program three two‐state DNA machines. (Reproduced with permission from Ref. [24], Copyright: Springer Nature (2010)).

**FIGURE 5:**
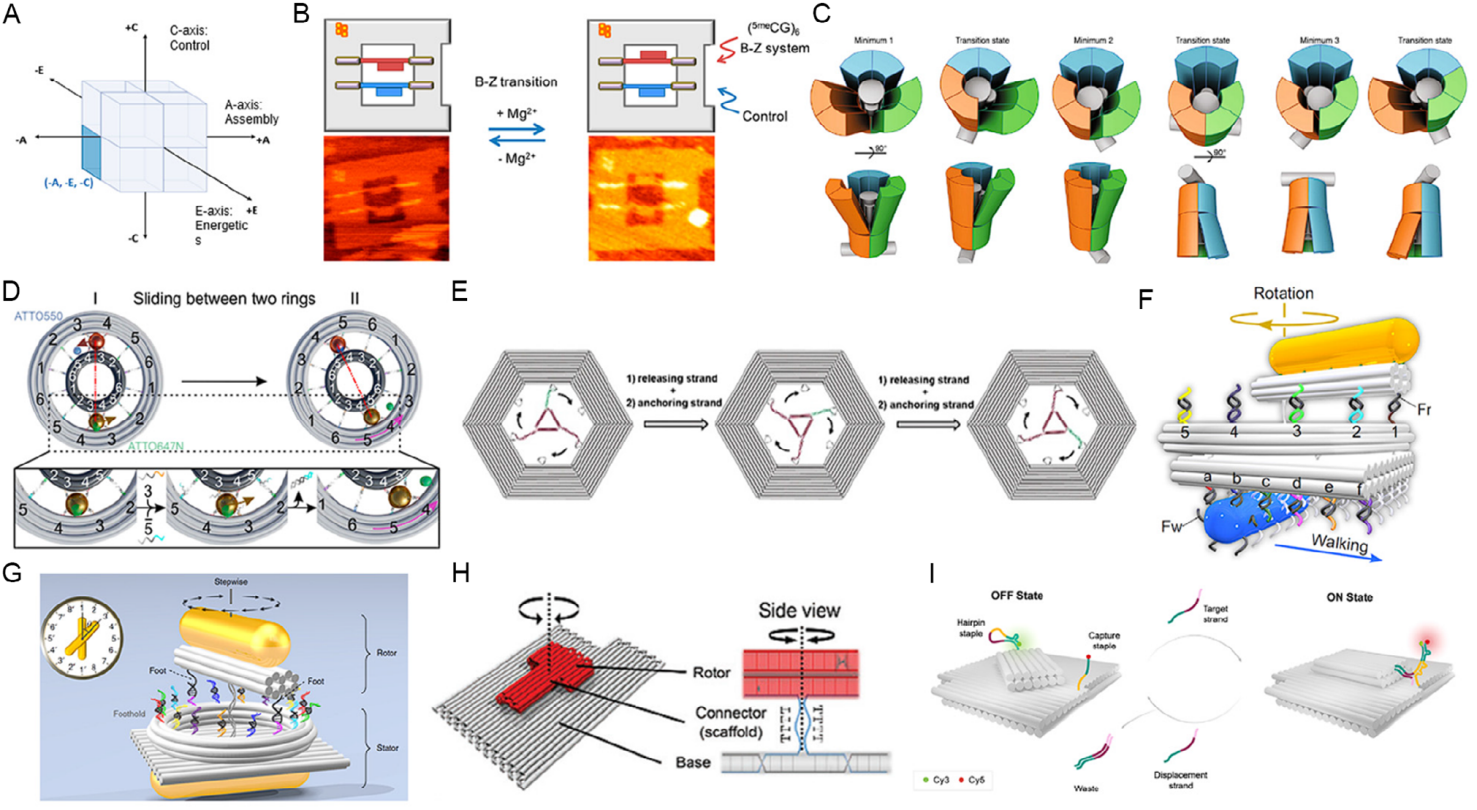
Representative Examples of the ‐Integrated Global (–A) ‐Passive (–E) ‐nonautonomous (–C) octant within the AEC Framework. (A) AEC Framework Conceptual Diagram: A cubic representation highlighting the three orthogonal axes of the AEC framework (Assembly, Energetics, Control). The highlighted area indicates the –A, –E, –C octant. (B) Rajendran et al. (2013) [27] Design: A rotary DNA nanomachine (–A) based on B‐Z conformational transition of DNA driven by external stimuli such as MgCl2 (–E), with nonautonomous control through addition of ions (–C). (Reproduced with permission from Ref. [28], Copyright: American Chemical Society (2012)) (C) Bertosin et al. (2021) [19] Design: A rotary mechanism with an asymmetric camshaft inside a stator (–A), driven by Brownian motion (–E), with nonautonomous control through mechanical stiffness changes via design variations (–C). (Reproduced with permission from Ref. [17], Copyright: Springer Nature (2021)) (D) Peil et al. (2022) [4] Design: A rotary planetary gearset nanodevice (–A) composed of modular DNA origami rings and gold nanoparticles, powered by DNA fuels via strand displacement reactions (–E), with nonautonomous control through sequential addition of fuels (–C). (Reproduced with permission from Ref. [5], Copyright: American Chemical Society (2022)) (E) Yang et al. (2019) [29] Design: A hexagonal DNA nanomachine with a three‐armed rotating nanostructure (–A), powered by DNA fuels via strand displacement reactions (–E), with nonautonomous control through sequential addition of fuels (–C). (Reproduced with permission from Ref. [30], Copyright: Wiley‐VCH (2019)) (F) Xin et al. (2021) [31] Design: Dimerization and oligomerization of DNA‐assembled building blocks with rotary and walking modules (–A), powered by DNA fuels via strand displacement reactions (–E), with nonautonomous control through sequential addition of fuels (–C). (Reproduced with permission from Ref. [32], Copyright: Springer Nature (2021)) (G) Xin et al. (2019) [33] Design: A rotary plasmonic nanoclock with a rotor gold nanorod (–A), powered by DNA fuels via strand displacement reactions (–E), with nonautonomous control through sequential addition of fuels (–C). (Reproduced with permission from Ref. [27], Copyright: Springer Nature (2019)) (H) Tomaru et al. (2017) [34] Design: A rotary DNA origami device with a rotor immobilized on a substrate (–A), powered by DNA fuels via strand displacement reactions (–E), with nonautonomous control through sequential addition of fuels (–C). (Reproduced with permission from Ref. [29], Copyright: Royal Society of Chemistry (2017)) (I) Tsang et al. (2026) [28] Design: A reconfigurable DNA origami rotary nanodevice comprising a flat sheet and a rotating top bundle (–A), powered by toehold‐mediated strand displacement reactions (–E), with nonautonomous control through the sequential addition of target and displacement strands for continuous nucleic acid sensing (–C). (Reproduced with permission from Ref. [28], Copyright: American Chemical Society (2026)).

**FIGURE 6:**
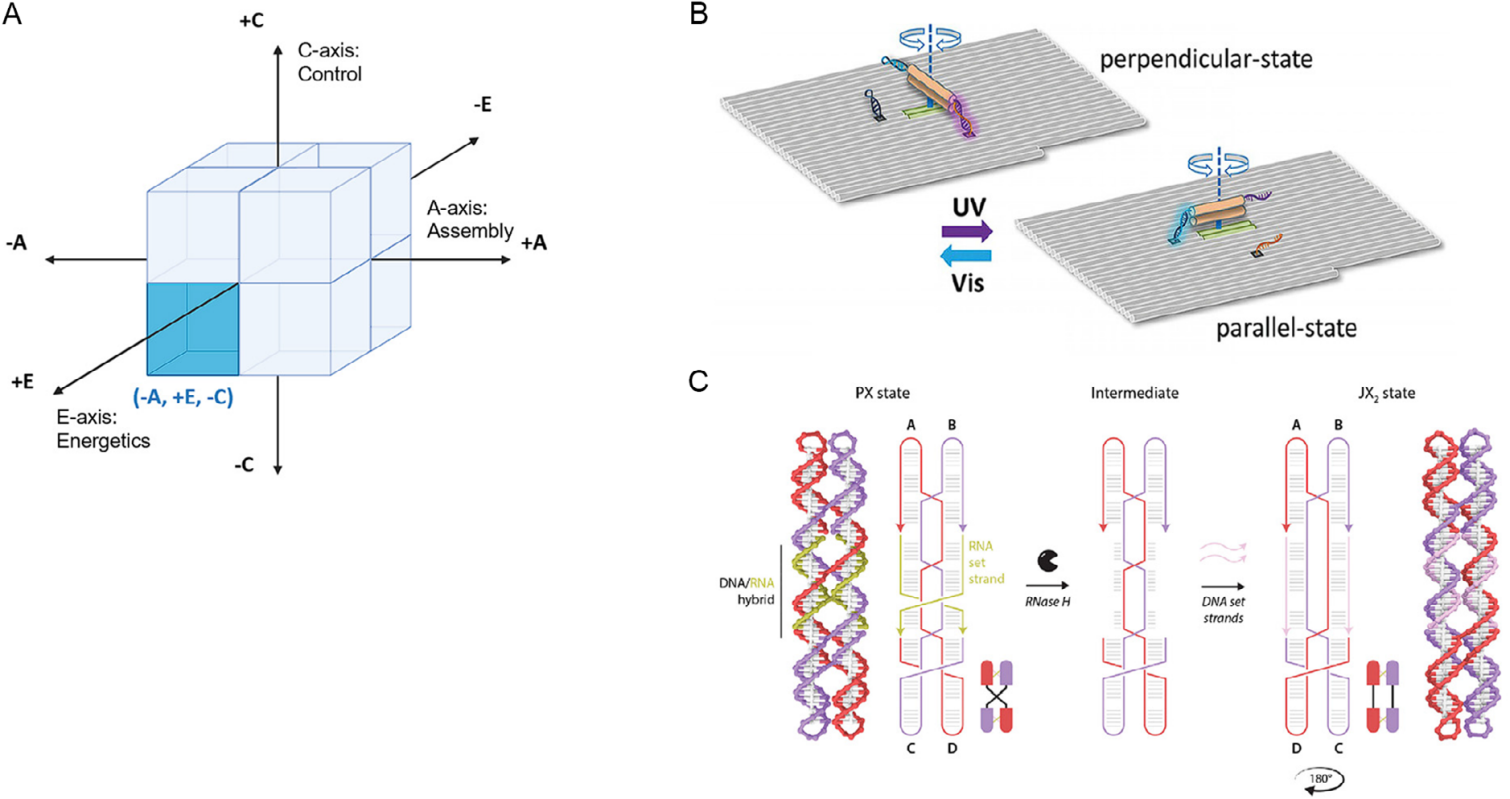
Representative Examples of the ‐Global (–A) +Active (+E) ‐nonautonomous (–C) octant within the AEC Framework. (A) AEC Framework Conceptual Diagram: A cubic representation highlighting the three orthogonal axes of the AEC framework (Assembly, Energetics, Control). The highlighted area indicates the –A, +E, ‐C octant. (B) Yang et al. (2017) [30] Design: A bar‐shaped double‐crossover rotor on a rectangular origami (–A), powered by photoactivation of azobenzene‐modified oligonucleotides for Active (+E) conformational switching, under nonautonomous (–C) control via alternating UV/visible light irradiation, achieving reversible rotation between perpendicular and parallel states visualized by high‐speed AFM. (Reproduced with permission from Ref. [21], Copyright: Wiley‐VCH (2017)). (C) Chandrasekaran (2024) [32] Design: A Paranemic crossover‐Juxtapose crossover_2_ DNA rotary nanodevice with RNA set strands, powered by RNase H enzymatic degradation for Active (+E) unsetting to a naked frame intermediate, under nonautonomous (–C) control via sequential enzyme and DNA strand addition for resetting, achieving 180° rotation of helical domains. (Reproduced with permission from Ref. [22], Copyright: Royal Society of Chemistry (2024)).

The AEC axes also clarify the deep coupling between energetics, control, and application goals. Biocompatibility, for instance, is a major driver. Passive (–E) systems are commonly paired with nonautonomous (–C) control, a synergy ideally suited for applications like drug delivery, where precise, gradual action and the avoidance of harsh chemical fuels are paramount. In the modular (+A) domain (Figure 4), systems typically rely on the sequential addition of fuel strands, as seen in the topology switches by Yan et al. [23] (Figure 4B), circular assemblies by Wang et al. [25] (Figure 4D), and nanoscale assembly lines by Gu et al. [26] (Figure 4E), or respond to environmental triggers like pH and ions as demonstrated by Lu et al. [24] (Figure 4C). Similarly, global (–A) architectures in the –A/–E/–C octant (Figure 5) frequently utilize stepwise DNA fuel additions (Figure 5D–H) or ion‐driven conformational changes (Figure 5B) to execute precise step‐by‐step movements, while Bertosin et al.'s camshaft [19] (Figure 5C) utilizes Brownian motion regulated by programmed structural stiffness. Pushing this specific regime toward advanced diagnostic applications, a recent breakthrough by Tsang et al. [28] (Figure 5I) presents a reconfigurable rotating nanodevice for continuous, single‐molecule nucleic acid sensing. By relying on the sequential addition of target and displacement strands to trigger toehold‐mediated rotational switching, this system brilliantly exemplifies how passive, nonautonomous global architectures can be harnessed for highly specific, multiplexed biomedical detection. However, Active (+E) systems can also operate under nonautonomous (–C) control, as exemplified by designs in the –A/+E/–C octant (Figure 6). Yang et al.'s photoactivated rotor [30] (Figure 6B) and Chandrasekaran's RNase H‐powered device (Figure 6C) demonstrate how external triggers (e.g. light or enzyme addition) enable controlled motion without full autonomy – a critical feature for applications requiring precise temporal regulation.

**FIGURE 7:**
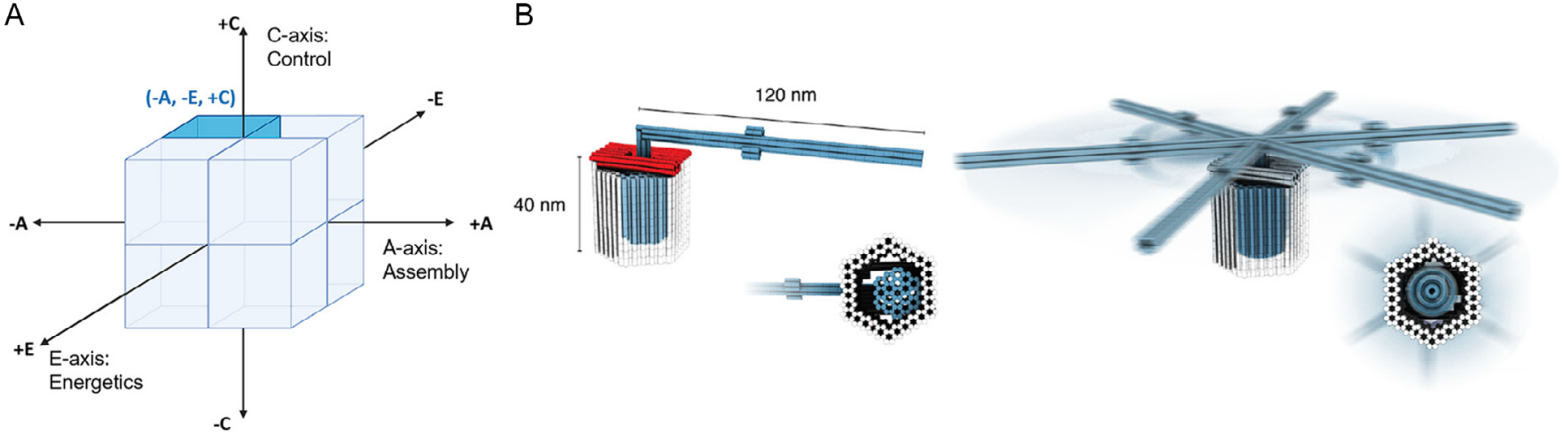
Representative Examples of the ‐Global (‐A) ‐Passive (‐E) +Autonomous (+C) octant within the AEC Framework. (A) AEC Framework Conceptual Diagram: A cubic representation highlighting the three orthogonal axes of the AEC framework (Assembly, Energetics, Control). The highlighted area indicates the ‐A, ‐E, +C octant. (B) Ketterer et al. (2016) [2] Design: A 3D DNA nanoscale rotor (blue) and stator (gray) complex (‐A), driven along the circumference by Brownian motion (‐E) and pausing at docking sites, whose intrinsic rotational freedom allows it to operate autonomously (+C). (Reproduced with permission from Ref. [3], Copyright: AAAS (2016)).

One notable exception is the design by Ketterer et al. [2] (Figure 7B), which harnesses the interplay of Brownian motion and docking sites to enable autonomous motion near thermal equilibrium. Autonomous (+C) control is essential for biocompatible tasks, such as intracellular mixing, and indispensable in complex environments where external triggers are impractical. This shift toward autonomy marks a fundamental evolution from mere mechanical efficiency to molecular intelligence – the capacity to sense, decide, and respond at the nanoscale.

Crucially, the framework's prediction lies in identifying sparsely populated octants that represent significant opportunities for engineering and scientific exploration. Notably, biological rotary nanomachines corresponding to nonautonomous (–C) control (e.g., the +A/+E/–C and +A/–E/–C octants) are virtually nonexistent, as nature relies on continuous self‐regulation rather than step‐by‐step external intervention. Therefore, exploring these biologically empty design spaces using DNA nanotechnology provides a unique opportunity to create artificial machines with on‐demand, programmable functionalities that diverge from, and elegantly complement, natural systems.

Beyond these nonautonomous domains, pushing the boundaries of fully autonomous systems across different assembly and energetic regimes presents critical engineering frontiers. For instance, global, actively powered, and autonomous (–A/+E/+C) architectures push the limits of raw mechanical precision and high‐force output required for mechanobiology. Conversely, modular, actively powered, and autonomous (+A/+E/+C) designs target the scalability needed to deploy programmable actuators in large‐scale environments, such as next‐generation DNA‐based microarrays and computing chips. Ultimately, mapping these diverse systems within the 3D design space reveals not only the strengths of current paradigms but also exposes the crucial gaps in the landscape. Systematically navigating this framework to bridge these populated frontiers with the completely unexplored octants will be the key to the next evolution of synthetic molecular machines.

## 4 Conclusion

The AEC framework provides a unified language rooted in engineering principles to classify, compare, and guide the development of rotary DNA nanomachines. Currently, distinct design philosophies dominate this space. Global (–A), actively powered (+E), autonomous (+C) machines achieve exceptional precision. Conversely, modular (+A), passive (‐E), nonautonomous (–C) devices prioritize scalability and precise manipulation at the molecular level.

The most transformative breakthroughs will emerge from the unexplored territories of this framework. For instance, the modular, passive, and autonomous domain (+A/‐E/+C) represents a highly coveted milestone, creating true artificial counterparts to natural enzymes that operate perpetually on ambient heat. Equally crucial is the modular, active, and externally controlled space(+A/+E/‐C), which paves the way for scalable, synchronized motor arrays with dynamic repair capabilities. It is also instructive to contextualize these rotary systems within the broader landscape of DNA nanomachines. While remarkable progress has been made in directional linear motors, such as the advanced walkers recently reported by Harashima et al. [35], rotary machines offer distinct geometric advantages. Unlike linear systems that require extended spatial tracks to operate, rotary architectures uniquely enable cyclic mechanical output within a strictly confined nanoscale footprint. This elimination of translational space makes them exceptionally well‐suited for high‐density integration into practical devices.

However, translating these conceptual frontiers into practical devices requires overcoming bottlenecks in physiological stability, torque efficiency, and large‐scale manufacturing. The framework facilitates this by isolating specific design dimensions for targeted innovation. Stability within the assembly dimension can be enhanced by scaling up to mesoscale superlattices or hybridizing with rigid inorganic nanoparticles. Efficiency within the energetic dimension can be driven by RNA polymerization, acoustic waves, or programmable plasmonics. Furthermore, complex feedback circuits in the control dimension can be automated using advanced computational tools, including predictive neural networks and transfer learning algorithms.

Looking ahead, future innovations will not only occupy these distinct spaces but also bridge them, leading to adaptive architectures that seamlessly shift between external commands and autonomous regulation. By understanding the deep correlation among assembly, energetics, and control, the next generation of DNA motors will transcend mere mechanical efficiency to achieve true molecular intelligence. These systems will possess the intrinsic capacity to sense, compute, and respond. Ultimately, this framework serves to guide the evolution of rotary DNA nanotechnology from elegant structural ingenuity toward robust, functional significance in emerging fields such as precision nanomedicine, synthetic cell design, and smart biomolecular computing.

## Author Contributions

**Xue‐Yan Wang:** conceptualization, writing ‐ review & editing, writing ‐ original draft. **Yicheng Heng**: visualization, writing ‐ original draft. **Julian A. Tanner:** writing ‐ review & editing, project administration. **Simon Chi‐Chin Shiu**: conceptualization, supervision, writing ‐ review & editing, writing ‐ original draft, project administration.

## Funding

This study was supported by Hong Kong University Grants Council General Research Fund (grant 17125920, 17125221, 17127124), Hong Kong University Grants Council Theme‐based Research Scheme (grant T12‐201/20‐R), HKU Seed Funding for Strategic Interdisciplinary Research (grant 102009959), HKU Seed Funding for Basic Research (grant 328279218).

## Conflicts of Interest

The authors declare no conflicts of Interest.

## References

[1] N. C. Seeman , “Nucleic Acid Junctions and Lattices,” Journal of Theoretical Biology 99 (1982): 237–247. 6188926 10.1016/0022-5193(82)90002-9

[2] P. Ketterer , E. M. Willner , and H. Dietz , “Nanoscale Rotary Apparatus Formed From Tight‐Fitting 3D DNA Components,” Science Advances 2 (2016): e1501209. 26989778 10.1126/sciadv.1501209 PMC4788491

[3] J. Valero , N. Pal , S. Dhakal , N. G. Walter , and M. Famulok , “A Bio‐Hybrid DNA Rotor‐Stator Nanoengine that Moves along Predefined Tracks,” Nature Nanotechnology 13 (2018): 496–503. 10.1038/s41565-018-0109-z PMC5994166 29632399

[4] A. Peil , L. Xin , S. Both , et al., “DNA Assembly of Modular Components into a Rotary Nanodevice,” ACS Nano 16 (2022): 5284–5291. 35286063 10.1021/acsnano.1c10160 PMC9047004

[5] R. R. Craig Jr and A. J. Kurdila , Fundamentals of Structural Dynamics (John Wiley & Sons, 2006).

[6] W. Beitz , G. Pahl , and K. J. M. B. Grote , “Engineering Design: A Systematic Approach,” Mrs Bulletin, 71 (1996) 30.

[7] T. Tørring , N. V. Voigt , J. Nangreave , H. Yan , and K. V. Gothelf , “DNA Origami: A Quantum Leap for Self‐Assembly of Complex Structures,” Chemical Society Reviews 40 (2011): 5636–5646. 21594298 10.1039/c1cs15057j PMC3328352

[8] C. Ma , W. Xu , W. Liu , C. Xu , W. Si , and J. Sha , “Precise Control of CNT‐DNA Assembled Nanomotor Using Oppositely Charged Dual Nanopores,” Nanoscale 15 (2023): 11052–11063. 37350160 10.1039/d3nr01912h

[9] K. I. Ito , Y. Sato , and S. Toyabe , “Design of Artificial Molecular Motor Inheriting Directionality and Scalability,” Biophysical Journal 123 (2024): 858–866. 38425042 10.1016/j.bpj.2024.02.026 PMC10995430

[10] H. Ramezani and H. Dietz , “Building Machines with DNA Molecules,” Nature Reviews. Genetics 21 (2020): 5–26. 10.1038/s41576-019-0175-6 PMC6976304 31636414

[11] X. Mao , M. Liu , Q. Li , C. Fan , and X. Zuo , “DNA‐Based Molecular Machines,” JACS Au 2 (2022). 2381–2399. 36465542 10.1021/jacsau.2c00292 PMC9709946

[12] S. M. Douglas , H. Dietz , T. Liedl , B. Högberg , F. Graf , and W. M. Shih , “Self‐Assembly of DNA into Nanoscale Three‐Dimensional Shapes,” Nature 459 (2009): 414–418. 19458720 10.1038/nature08016 PMC2688462

[13] E. Winfree , F. Liu , L. A. Wenzler , and N. C. Seeman , “Design and Self‐Assembly of Two‐Dimensional DNA Crystals,” Nature 394 (1998): 539–544. 9707114 10.1038/28998

[14] P. W. Rothemund , “Folding DNA to Create Nanoscale Shapes and Patterns,” Nature 440 (2006): 297–302. 16541064 10.1038/nature04586

[15] A. K. Pumm , W. Engelen , E. Kopperger , et al., “A DNA Origami Rotary Ratchet Motor,” Nature 607 (2022): 492–498. 35859200 10.1038/s41586-022-04910-y PMC9300469

[16] X. Shi , A. K. Pumm , C. Maffeo , et al., “A DNA Turbine Powered by a Transmembrane Potential across a Nanopore,” Nature Nanotechnology 19 (2024): 338–344. 10.1038/s41565-023-01527-8 PMC10950783 37884658

[17] M. Endo and H. Sugiyama , “Chemical Approaches to DNA Nanotechnology,” Chembiochem : A European Journal of Chemical Biology 10 (2009): 2420–2443. 19714700 10.1002/cbic.200900286

[18] F. C. Simmel , B. Yurke , and H. R. Singh , “Principles and Applications of Nucleic Acid Strand Displacement Reactions,” Chemical Reviews 119 (2019): 6326–6369. 30714375 10.1021/acs.chemrev.8b00580

[19] E. Bertosin , C. M. Maffeo , T. Drexler , M. N. Honemann , A. Aksimentiev , and H. Dietz , “A Nanoscale Reciprocating Rotary Mechanism with Coordinated Mobility Control,” Nature Communications 12 (2021): 7138. 10.1038/s41467-021-27230-7 PMC8654862 34880226

[20] B. Yurke , A. J. Turberfield , F. C. Simmel , A. P. Mills Jr. , and J. L. Neumann , “A DNA‐Fuelled Molecular Machine Made of DNA,” Nature 406 (2000): 605–608. 10949296 10.1038/35020524

[21] P. Kosuri , B. D. Altheimer , M. Dai , P. Yin , and X. Zhuang , “Rotation Tracking of Genome‐Processing Enzymes Using DNA Origami Rotors,” Nature 572 (2019): 136–140. 31316204 10.1038/s41586-019-1397-7 PMC7036295

[22] K. E. Dunn , M. C. Leake , A. J. Wollman , M. A. Trefzer , S. Johnson , and A. M. Tyrrell , An Experimental Study of the Putative Mechanism of a Synthetic Autonomous Rotary DNA Nanomotor,” Royal Society open science 4 (2017): 160767. 28405363 10.1098/rsos.160767 PMC5383820

[23] H. Yan , X. Zhang , Z. Shen , and N. C. Seeman , “A Robust DNA Mechanical Device Controlled by Hybridization Topology,” Nature 415 (2002): 62–65. 11780115 10.1038/415062a

[24] C. H. Lu , A. Cecconello , J. Elbaz , A. Credi , and I. Willner , “A Three‐Station DNA Catenane Rotary Motor with Controlled Directionality,” Nano Letters 13 (2013): 2303–2308. 23557381 10.1021/nl401010e

[25] L. Wang , Z. Meng , F. Martina , H. Shao , and F. Shao , “Fabrication of Circular Assemblies with DNA Tetrahedrons: From Static Structures to a Dynamic Rotary Motor,” Nucleic Acids Research 45 (2017): 12090–12099. 29126166 10.1093/nar/gkx1045 PMC5716610

[26] H. Gu , J. Chao , S. J. Xiao , and N. C. Seeman , “A Proximity‐Based Programmable DNA Nanoscale Assembly Line,” Nature 465 (2010): 202–205. 20463734 10.1038/nature09026 PMC2872101

[27] A. Rajendran , M. Endo , K. Hidaka , and H. Sugiyama , “Direct and Real‐Time Observation of Rotary Movement of a DNA Nanomechanical Device,” Journal of the American Chemical Society 135 (2013): 1117–1123. 23311576 10.1021/ja310454k

[28] E. Tsang , L. M. Lund , V. Birkedal , and K. V. Gothelf , “Single‐Molecule Nucleic Acid Detection with a Reconfigurable Rotating DNA Origami Nanodevice,” ACS Nano 20 (2026): 4607–4616. 41586571 10.1021/acsnano.5c22080

[29] Y. Yang , S. Zhang , S. Yao , et al., “Programming Rotary Motions with a Hexagonal DNA Nanomachine,” Chemistry‐‐A European Journal 25 (2019): 5158–5162. 30791173 10.1002/chem.201900221

[30] Y. Yang , R. Tashiro , Y. Suzuki , et al., “A Photoregulated DNA‐Based Rotary System and Direct Observation of Its Rotational Movement,” Chemistry 23 (2017): 3979–3985. 28199775 10.1002/chem.201605616

[31] L. Xin , X. Duan , and N. Liu , “Dimerization and Oligomerization of DNA‐Assembled Building Blocks for Controlled Multi‐Motion in High‐Order Architectures,” Nature Communications 12 (2021): 3207. 10.1038/s41467-021-23532-y PMC8163789 34050157

[32] A. R. Chandrasekaran , A DNA Rotary Nanodevice Operated by Enzyme‐Initiated Strand Resetting, Chemical Communications 60 (2024). 534–537, 38038977 10.1039/d3cc05487j PMC10843534

[33] L. Xin , C. Zhou , X. Duan , and N. Liu , “A Rotary Plasmonic Nanoclock,” Nature Communications 10 (2019): 5394. 10.1038/s41467-019-13444-3 PMC6881389 31776340

[34] T. Tomaru , Y. Suzuki , I. Kawamata , S. M. Nomura , and S. Murata , “Stepping Operation of a Rotary DNA Origami Device,” Chemical Communications 53 (2017): 7716–7719, 28548145 10.1039/c7cc03214e

[35] T. Harashima , A. Otomo , and R. Iino , “Rational Engineering of DNA‐Nanoparticle Motor with High Speed and Processivity Comparable to Motor Proteins,” Nature Communications 16 (2025): 729. 10.1038/s41467-025-56036-0 PMC11739693 39820287

